# Adherence to the Dietary Approaches to Stop Hypertension diet reduces the risk of breast cancer: A systematic review and meta-analysis

**DOI:** 10.3389/fnut.2022.1032654

**Published:** 2023-01-09

**Authors:** Long Shu, Yi-Qian Huang, Xiao-Yan Zhang, Pei-Fen Zheng, Qin Zhu, Jian-Ying Zhou

**Affiliations:** ^1^Department of Nutrition, Zhejiang Hospital, Hangzhou, Zhejiang, China; ^2^Department of Digestion, Zhejiang Hospital, Hangzhou, Zhejiang, China

**Keywords:** breast cancer, Dietary Approaches to Stop Hypertension, DASH diet, systematic review, meta-analysis, epidemiology

## Abstract

**Background:**

Despite increasing evidence for the association of adherence to the Dietary approaches to stop hypertension (DASH) diet with breast cancer risk, the results remain inconclusive. The purpose of the current systematic review was to summarize the evidence from previous observational studies and explore the potential association between DASH diet and breast cancer risk using meta-analysis.

**Methods:**

A comprehensive literature search was conducted using the databases of PubMed, Web of Science, CNKI and Wanfang Data to identify the relevant publications from inception up to July 2022. The pooled relative risks (RRs) and 95% confidence intervals (CIs) were calculated for the highest versus the lowest categories of DASH score in relation to breast cancer risk, using a random-effects model. The Cochran’s *Q* test and I-squared (*I*^2^) statistic were used to detect the sources of heterogeneity among the included studies.

**Results:**

Overall, eleven studies, involving 23,254 breast cancer cases and 449,273 participants, were included in this systematic review and meta-analysis. Combining 16 effect sizes from 11 studies, a significant inverse association between adherence to the DASH diet and risk of breast cancer was observed (RR = 0.79; 95% CI: 0.70, 0.90, *P* < 0.0001). Stratified analysis showed a significant association between adherence to the DASH diet and risk of breast cancer in case-control studies (RR = 0.49; 95% CI: 0.27–0.89, *P* = 0.019), and a marginally significant association in prospective cohort studies (RR = 0.92; 95% CI: 0.86–0.98, *P* = 0.014), respectively. Besides, a more significant association between DASH score and reduced risk of breast cancer was observed in Asian countries (RR = 0.50; 95% CI: 0.31–0.81, *P* = 0.005) than in the United States (RR = 0.93; 95% CI: 0.89–0.99, *P* = 0.012). Similarly, when we conducted analyses separately by menopausal status, we found a significant inverse association between DASH diet and breast cancer risk in postmenopausal women (RR = 0.58; 95% CI: 0.39–0.87, *P* = 0.008).

**Conclusion:**

The results of this systematic review and meta-analysis indicate a significant inverse association between adherence to the DASH diet and risk of breast cancer. Further large prospective studies and randomized controlled trials are required to confirm our findings.

## 1. Introduction

Breast cancer is the most common malignancy among women worldwide, and the annual incidence rates continue to rise (1). According to the estimates from the International Agency for Research on Cancer (IARC) in 2020, female breast cancer has surpassed lung cancer as the leading cause of global cancer incidence, with an estimated 2.3 million new cases, accounting for 11.7% of all cancer cases (2). Although the incidence rate of breast cancer is lower in China than in Western countries, its incidence and mortality rate has risen substantially in last decades (3). Of note, breast cancer is the fourth most commonly diagnosed cancer in China, with approximately 0.42 million new cases in 2020 (2). Indeed, this ongoing rising trend reflects the necessity of urgency for implementing effective prevention strategies. Multiple risk factors contribute to increased risk of breast cancer including alcohol intake, obesity, a sedentary lifestyle, family history, menstrual and reproductive history, exogenous hormone intake and never giving birth or breastfeeding have been well established (4). Along with these aforementioned risk factors, diet factors have been identified as important and modifiable risk factors in the development of breast cancer (5).

The Dietary Approaches to Stop Hypertension (DASH) diet which emphasizes high intakes of fruits, vegetables, whole grains, nuts and legumes, moderate intakes of low-fat dairy products, and low intakes of sodium, sugar-sweetened beverages and red/processed meats, is initially designed to counteract high blood pressure (6). In contrast to usual diets, this pattern may provide higher amounts of potassium, calcium, magnesium, fiber, and protein, and lower amounts of sodium, saturated fat and dietary cholesterol. Currently, it has already been recommended as a healthy dietary guideline for the general public by the United States Department of Agriculture (7). Later investigations have observed that adopting the DASH diet may beneficially affect several non-communicable diseases, such as cardiovascular diseases, chronic kidney disease and some types of cancer (8–12). Of note, the effects of the DASH diet on breast cancer have been less studied.

In the last decade, the relationship between DASH diet and breast cancer has been a concern for researchers. To date, numerous observational studies have attempted to explore the association between adherence to the DASH diet and breast cancer incidence and mortality (13–17). However, the results of theses studies are not entirely consistent. While several studies have shown a significant inverse relationship between adherence to the DASH diet and breast cancer risk (15, 16), other studies exhibited the null association (13, 15). Meanwhile, in the World Cancer Research Fund/American Institute for Cancer Research (WCRF/AICR) 2007 report, no firm judgment was made on the possible relationship between plant-based dietary patterns (characterized by higher intake of plant foods and lower intake of animal foods) and risk of breast cancer (18). Furthermore, according to our knowledge, the relationship between DASH diet and breast cancer has not been studied yet in a systematic review and meta-analysis. Therefore, to identify the potential association between adherence to the DASH diet and risk of breast cancer, we carried out this systematic review and meta-analysis of observational studies published from inception up to July 2022.

## 2. Materials and methods

We followed the Meta-Analysis of Observational Studies in Epidemiology (MOOSE) and Preferred Reporting Items for Systematic Reviews and Meta-Analysis (PRISMA) guidelines for reporting this study (19).

### 2.1. Literature search strategy

An electronic literature search *via* four databases, including PubMed, Web of Science, CNKI, and Wanfang Data was conducted to identify relevant articles written in the English or Chinese languages published from their dates of inception up to July 2022, with the following terms: {[“breast adenoma” (all fields) OR “breast carcinoma” (all fields) OR “breast cancer” (all fields) OR “breast tumor” (all fields) OR “breast neoplasms” (all fields) OR “breast neoplasms” (MeSH)] AND [“DASH score” (all fields) OR “DASH diet” (all fields) OR “DASH” (all fields) OR “Dietary Approaches To Stop Hypertension” (all fields) OR “Dietary Approaches To Stop Hypertension” (MeSH)]}. Additionally, we also manually searched all references lists of retrieved articles and previously published reviews to identify potentially eligible studies. All of these steps were accomplished by two independent reviewers (LS and Y-QH), and any disagreements with article selection were resolved through discussion with QZ.

### 2.2. Studies included criteria

Two reviewers (LS and J-YZ) independently screened the titles and abstracts of articles retrieved in the initial search to identify studies that examined the association between DASH diet and breast cancer risk. Any disagreements were settled by discussion or in consultation with the third reviewer. When all agreed, the full-text versions of articles were reviewed against inclusion and exclusion criteria for this systematic review and meta-analysis. To be eligible for inclusion, studies met the following criteria: (1) evaluated the association between DASH diet and breast cancer risk in an observational study; (2) estimated odds ratios (ORs), hazard ratios (HRs), or relative risk (RRs) along with their corresponding 95% confidence interval (CI) (or sufficient data to calculate them); (3) If the data in retrieved article lacked sufficient detail, the corresponding author of the original study was contacted by email; and (4) breast cancer diagnoses were confirmed by clinical interviews, or self-report on a previous physician-made diagnosis of breast cancer. If the same dataset had been published more than once, we selected the study with the largest number of participants or the best complete findings. Studies were excluded if they met the following criteria: (1) they were non-English or non-Chinese studies; (2) they were reviews, case reports, conference papers, letters, editorials, cellular and molecular studies, and animal studies (3) for studies based on the same cohort, we selected only the study with the most complete data. In total, eleven articles reported the association between DASH diet and breast cancer risk.

### 2.3. Data extraction

From the selected studies, we extracted the following information: the first author’s last name, publication date, country, study design, follow-up period, sample size, mean age/age range for cases and participants, the number of breast cancer cases, reported risk estimates (HR/OR/RR) and their corresponding 95% CI, and the factors that were adjusted for in the analysis. In the case of presenting menopausal status-stratified or effect sizes, we treated them as two separate studies. Besides, for a study that reported several risk estimates, the adjusted model was selected. All aforementioned steps were carried out by two reviewers, independently.

### 2.4. Quality assessment of included studies

The Newcastle-Ottawa Scale (20) was used to identify the quality of included studies in the meta-analysis. This scale consisted of three main domains: selection of participants with maximum 4 stars, comparability of participants with maximum 2 stars, and assessment of outcome/exposure with maximum 3 stars. The maximum score that a study can get is 9 and these studies with higher than 7 scores can be identified as high-quality (21). Differences were resolved by consensus with a third author (P-FZ).

### 2.5. Statistical analysis

Log-transformed RRs with their corresponding standard errors (SEs) were obtained using risk ratios (ORs, HRs, and RRs and corresponding 95% CIs) which were previously extracted for the association between adherence to the DASH diet and risk of breast cancer. Between-study heterogeneity was measured by Cochran’s *Q* test and *I*^2^ statistic. A *P*-value of *Q*-test > 0.10 or *I*^2^ < 50% indicated an absence of heterogeneity between studies, and a fixed-effects model was implemented to calculate the pooled RRs. If a *P*-value of *Q*-test ≤ 0.10 or *I*^2^ ≥ 50% indicated a high degree of heterogeneity among studies, then a random-effects model (DerSimonian and Laird method) was used (22). If there was substantial heterogeneity, the potential sources of heterogeneity were examined by using sensitivity and subgroup analyses. Subgroup analyses were performed based on study design (prospective cohort/case-control studies), menopause status (both/premenopausal/postmenopausal), country (United States/Asian countries), and comparison (Q5 vs. Q1/Q4 vs. Q1/Q3 vs. Q1). Publication bias was checked through the visual inspection of the funnel plot and quantified by the Begg’s test and Egger’s test (23). Sensitivity analyses were performed, excluding one study at a time to clarify whether the results were robust or sensitive to the influence of a single study. All statistical analyses were conducted with STATA, version 12 (StataCorp, College Station, TX, USA). A two-tailed *P* < 0.05 was considered to be statistically significant.

## 3. Results

### 3.1. Search results

**FIGURE 1:**
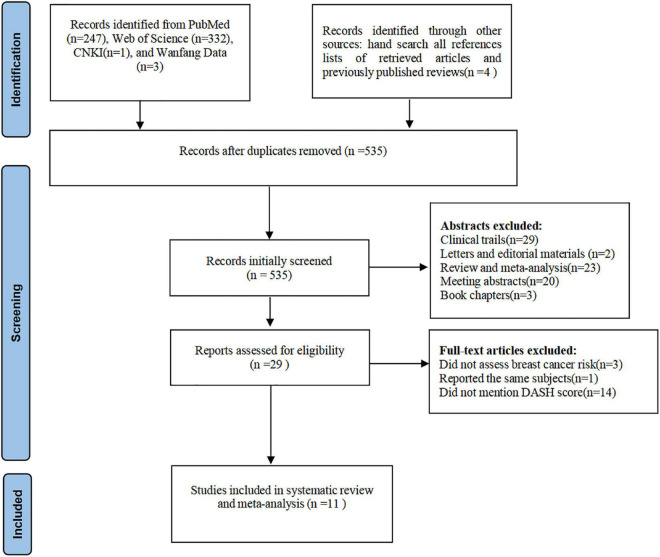
Flow chart of article screening and selection process.

The initial search yielded 587 potential articles, and after removing 52 duplicates, 535 articles were considered for screening. Reviewing titles and abstracts led to the exclusion of 506 studies because they did not report the association between DASH diet and risk of breast cancer, and the full-text versions of the remaining 29 articles were carefully checked for eligibility. Of the remaining 29 articles, 18 articles were excluded based on the following reasons: 3 did not assess breast cancer risk; 1 reported the same participants; 14 did not mention DASH score. Finally, eleven studies with 449,273 participants and 23,254 cases of breast cancer were selected for the final analysis (12–16, 23–28). Flow chart of article screening and selection process are depicted in Figure 1.

### 3.2. Study characteristics

The characteristics of all included studies assessing the association between adherence to DASH diet and breast cancer risk are shown in Table 1. Eight prospective cohort (14–16, 24–26, 28, 29) and three case-control (13, 17, 27) studies met the inclusion criteria and were included in this systematic review. Publication dates of these studies varied between 2013 and 2022. Seven of the included studies were carried out in the United States (14, 15, 24–26, 29), three in Iran (13, 17, 27), and one study in China (28). Age of participants ranged from ages 19 to 104 years. For exposure assessment, all included studies used FFQs. Besides, all included publications used methods designed by Fung et al. (7 food groups and sodium) (14–17, 24–30), and Dixon et al. (7 food groups, saturated fat and alcohol) (13, 31) to extracted DASH diet. For outcome assessment, four studies had used cancer registries (15, 26, 28, 29), five studies used medical records (13, 14, 16, 17, 24), and two studies used pathology reports (25, 27). Out of all included studies, four studies reported the significant inverse relationship between DASH diet and risk of breast cancer (13, 16, 17, 28), while seven studies found no statistical association in this regard (14, 15, 24–27, 29).

**TABLE 1:** Characteristics of the included studies on the association between DASH diet and risk of breast cancer.

References	Study design	Country	Sample size	Follow-up (years)	Mean age/Age range	Exposure assessment	Outcome assessment	Study quality	Adjustment
Tangestani et al. (12)	Case-control	Iran	132 cases/272 controls	–	≥30 years	FFQ Self-report	Medical records	6	Age, BMI, energy intake, physical activity, age at first live birth, vitamin D supplements and family history of cancer
Heidari et al. (13)	Cohort	USA	4103 (453 cases)	18 years	30–55 years	FFQ Self-report	Medical records	8	Age at diagnosis (years), quintiles of energy intake, body mass index, body mass index change, age at first birth and parity, oral contraceptive use, menopausal status and HRT use, smoking, stage of disease, radiation treatment, chemotherapy and hormonal treatment, and physical activity
Izano et al. (14)	Cohort	USA	101291 (7749 cases)	17.4 years	45–75 years	FFQ Self-report	Registries	8	Age, total energy intake, BMI, smoking status, physical activity, education, age at menarche, age at first live birth, parity, age at menopause, family history of breast cancer, estrogen and progestin use, diet quality index depending on the model and alcohol intake
Dela Cruz et al. (15)	Cohort	USA	50084 (1700 cases)	7.6 years	35–74 years	FFQ interview	Medical records	9	Total energy intake, race/ethnicity, income, smoking, BMI, physical activity, height, education, alcohol intake, mother diagnosed with breast cancer, age at first live birth, parity, hormone replacement therapy, age at menopause, oral contraception
Petimar et al. (16)	Case-control	Iran	350 cases/700 controls	–	≥30 y	FFQ interview	Medical records	7	Age, energy intake, education, residency, family history of breast cancer, physical activity, marital status, smoking, alcohol consumption, supplement use, breast-feeding, menopausal status and BMI
Begg and Mazumdar (23)	Cohort	USA	3660 (621 cases)	2.3 years	24–94 years	FFQ Self-report	Medical records	7	Age at diagnosis, total energy, race and ethnicity, education, menopausal status, physical activity, smoking, cancer stage, estrogen-receptor status, progesterone-receptor status, HER2, body mass index, surgery type, chemotherapy, radiation, and hormonal therapies
Ergas et al. (24)	Cohort	USA	86620 (5522 cases)	26 years	30–55 years	FFQ Self-report	Pathology records	9	Age, energy intake, multivitamin use, smoking, body mass index, height, weight at 18 years of age, weight change since 18 years of age, family history of breast cancer, benign breast disease, physical activity level, alcohol intake, and menopausal hormone use
Fung et al. (25)	Cohort	USA	96959 (3869 cases)	16 years	22–104 years	FFQ Self-report	Registries	9	Age at baseline, race, breast cancer family history, age at menarche, oral contraceptive use, parity status, smoking status, socioeconomic status, physical activity, total energy intake, and total alcohol intake, BMI
Haridass et al. (26)	Case-control	Iran	486 cases/523 controls	–	19–80 years	FFQ interview	Pathology records	6	Age, energy intake, education, smoking, alcohol intake, physical activity, family history of breast cancer, marital status, oral contraceptive use, parity, fertility treatment, hormone replace therapy, BMI.
Toorang et al. (27)	Cohort	China	3450 (456 cases)	5.3 years	25–70 years	FFQ Self-report	Registries	7	Age in 60-month survey, intervals between diagnosis and 60-month survey, total energy intake, income, education, marriage, menopausal status at diagnosis, BMI at 60-month survey, physical activity at 60-month survey, ER, PR, HER2, TNM stages, comorbidity, chemotherapy, radiotherapy and immunotherapy.
Wang et al. (28)	Cohort	USA	100643 (2372 cases)	22 years	30–55 years	FFQ Self-report	Registries	9	BMI at age 18, weight change since age 18 in kgs, physical activity in MET hours/week, energy intake in kilocalories/day, parity/age at first birth, menopausal hormone use, alcohol intake, oral contraceptive use, age at menarche, age at menopause, family history of breast cancer, and benign breast disease diagnosis.

DASH, Dietary Approaches to Stop Hypertension; FFQ, food frequency questionnaire; BMI, body mass index; USA, United States; HER2, human epidermal growth factor 2; ER, estrogen receptor; PR, progesterone receptor; MET, metabolic equivalent task.

### 3.3. Adherence to the DASH diet and breast cancer

**FIGURE 2:**
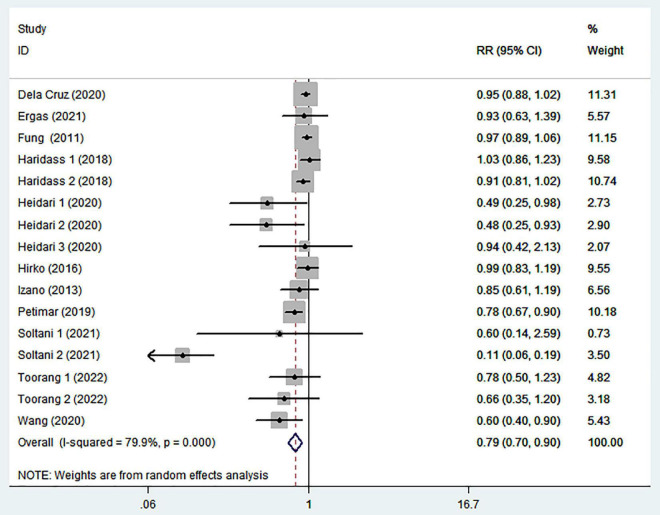
Meta-analysis on the association between adherence to dietary approaches to stop hypertension (DASH) diet and breast cancer risk.

The association between the highest intake compared with the lowest intake categories of DASH diet with breast cancer risk is shown in Figure 2. Combining 16 effect sizes from 11 studies (13–17, 24–29), we observed an inverse association between DASH diet and the risk of breast cancer (RR = 0.79; 95% CI: 0.70–0.90; *P* < 0.0001). The heterogeneity among the included studies was high (*P* < 0.00001; *I*^2^ = 79.9%), and hence the effect was assessed using the random-effects model.

### 3.4. Subgroup analyses

**FIGURE 3:**
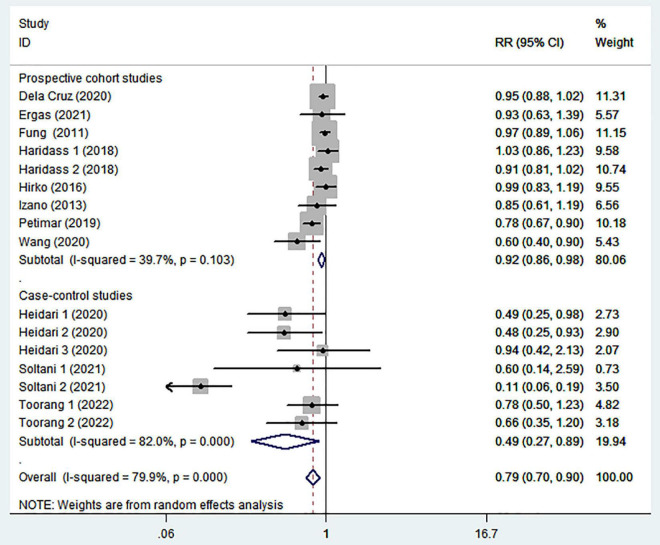
Meta-analysis on the association between adherence to dietary approaches to stop hypertension (DASH) diet and breast cancer risk stratified by studied design.

**FIGURE 4:**
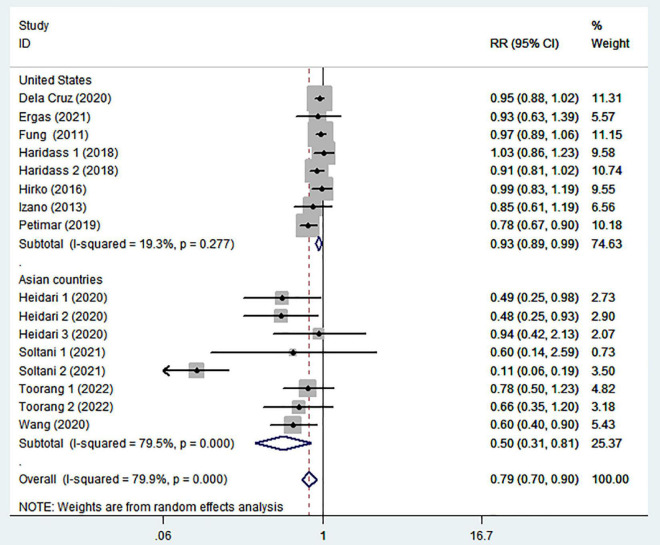
Meta-analysis on the association between adherence to dietary approaches to stop hypertension (DASH) diet and breast cancer risk stratified by country.

**FIGURE 5:**
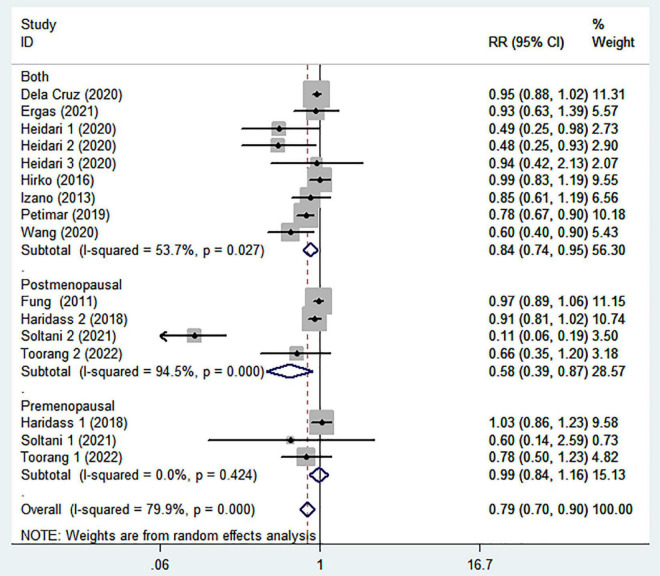
Meta-analysis on the association between adherence to dietary approaches to stop hypertension (DASH) diet and breast cancer risk stratified by menopausal status.

**FIGURE 6:**
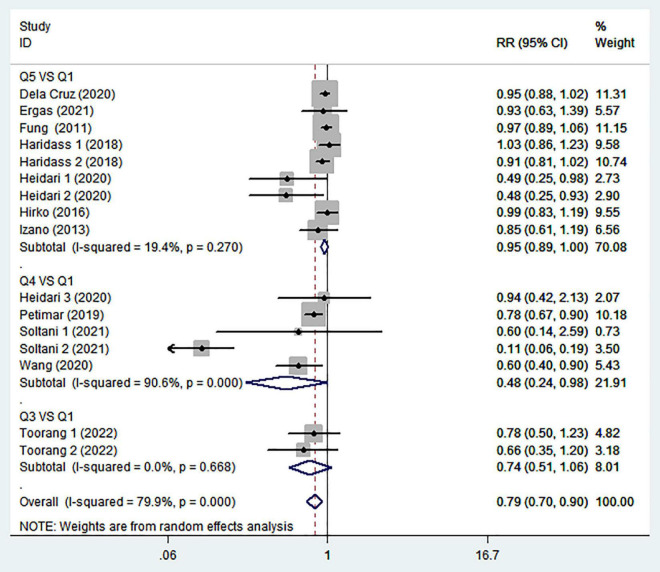
Meta-analysis on the association between adherence to dietary approaches to stop hypertension (DASH) diet and breast cancer risk stratified by comparison.

To further explore the reasons for heterogeneity among included studies, we carried out subgroup analyses according to study design, country, menopausal status, and comparison (Table 2). When we conducted analyses separately by study design (Figure 3), we found a significant inverse association between adherence to the DASH diet and breast cancer risk in case-control studies (RR = 0.49; 95% CI: 0.27–0.89, *P* = 0.019). However, the between-study heterogeneity was most apparent (*P* < 0.00001; *I*^2^ = 80%). In the prospective cohort studies, there was less evidence of heterogeneity (*P* = 0.103; *I*^2^ = 39.7%), and a marginally significant association between DASH diet and risk of breast cancer (RR = 0.92; 95% CI: 0.86–0.98, *P* = 0.014). The stratified association between DASH diet and risk of breast cancer according to country based on the random-effects model is provided in Figure 4. There was significant heterogeneity in Asian countries, where a decreased risk of breast cancer was shown (RR = 0.50; 95% CI: 0.31–0.81, *P* = 0.005). In United States, there was less evidence of heterogeneity (*P* = 0.277; *I*^2^ = 19.3%), and a small decrease in the risk of breast cancer was shown (RR = 0.93; 95% CI: 0.89–0.99, *P* = 0.012). When we conducted analyses separately by menopausal status (Figure 5), we found a significant inverse association between DASH diet and breast cancer risk in both and postmenopausal women (for both: RR = 0.84; 95% CI: 0.74–0.95, *P* = 0.007 and for postmenopausal: RR = 0.58; 95% CI: 0.39–0.87, *P* = 0.008). However, the heterogeneity was most apparent in postmenopausal women (*P* < 0.0001; *I*^2^ = 94.5%). Similarly, we also performed stratified analysis based on comparison in Figure 6. Among Q4 vs. Q1 comparison studies only, there was more heterogeneity (*P* < 0.00001, *I*^2^ = 90.6%), and a significantly decreased risk of breast cancer was shown (RR = 0.48; 95% CI: 0.24, 0.98; *P* = 0.045). In Q5 vs. Q1 comparison studies, there was no evidence of significant heterogeneity between studies (*P* = 0.270, *I*^2^ = 19.4%), and a marginally significant association between DASH diet and risk of breast cancer was shown (RR = 0.95; 95% CI: 0.89–1.00; *P* = 0.059).

**TABLE 2:** Subgroup analyses for the association between DASH diet and risk of breast cancer.

Study characteristic	Category	No. of studies	*I* ^2^	RR (95% CI)	*P*
Comparison	Q5 vs. Q1	7	19.4%	0.95 (0.89, 1.00)	0.270
	Q4 vs. Q1	4	90.6%	0.48 (0.24, 0.98)	<0.0001
	Q3 vs. Q1	1	0%	0.74 (0.51,1.06)	0.668
Menopausal status	Both	7	53.7%	0.84 (0.74, 0.95)	0.027
	Premenopausal	3	79.9%	0.99 (0.84, 1.16)	<0.0001
	Postmenopausal	4	94.5%	0.58 (0.39,0.87)	<0.0001
Country	United States	7	19.3%	0.93 (0.89, 0.99)	0.277
	Asian countries	4	79.5%	0.50 (0.11, 0.81)	<0.0001
Study design	Case-control	3	82.0%	0.49 (0.27, 0.89)	<0.0001
	Cohort	8	39.7%	0.92 (0.86, 0.98)	0.103

DASH, Dietary Approaches to Stop Hypertension; RR, relative risk; CI, confidence interval.

### 3.5. Publication bias

Funnel plots showed little evidence of asymmetry (Supplementary Figure 1) and therefore little evidence of publication bias (highest compared with lowest category of DASH diet: Begg’s test, *P* = 0.065).

### 3.6. Quality assessment

The quality of each study in terms of population and sampling methods, description of exposure and outcomes, and statistical adjustment of data, was shown in Appendix 1. When all included studies received a score of seven or higher, they would be deemed to be of relatively higher quality (14–17, 24–26, 28, 29).

### 3.7. Sensitivity analyses

Sensitivity analyses were performed by excluding each study individually to investigate the influence on overall estimates. Based on the results of sensitivity analysis (Supplementary Figure 2), no significant changes in the risk estimates were observed after removal of any single or a few studies in the case of the DASH diet in respect to risk of breast cancer.

## 4. Discussion

To the best of our knowledge, this is the first systematic review and meta-analysis summarizing the published evidence regarding the effect of adherence to the DASH diet on breast cancer. Data from eleven studies involving 23,254 breast cancer cases and 449,273 participants, were included in the present meta-analysis. Our results indicated that high adherence to the DASH diet was associated with a 21% reduction in the risk of breast cancer. Sensitivity analysis revealed that the summary effect of DASH diet on breast cancer was not substantially modified by excluding a certain study. Collectively, our findings augment the evidence for an inverse association between DASH diet and breast cancer risk, and support the adoption of adherence to the DASH diet for the primary prevention of breast cancer.

Among women, breast cancer accounts for 1 in 4 cancer cases and for 1 in 6 cancer deaths, ranking first for incidence in many countries (2). With an estimated 2.3 million new cases and 685,000 deaths worldwide, breast cancer has become the first most commonly diagnosed cancer and the fifth leading cause of cancer mortality in 2020 (2). This ongoing rising trend reflects the necessity of urgency for preventive measure. Among the main risk factors, dietary factors have garnered considerable attention (4). A substantial amount of epidemiological studies have been conducted to assess the impact of diet, a key modifiable risk factor on breast cancer risk (5, 32–35). However, the majority of these studies have largely focused on the effect of the intakes of individual nutrients, foods or food groups (32–34). Obviously, food and nutrients are never eaten in isolation and their effects are likely to interact (36). Thus, dietary patterns, which represent whole-diet and possible food and nutrient interactions, have been linked to the risk of breast cancer. In nutritional epidemiology, dietary patterns have been defined by several statistical methods, which can be distinguished as *a priori* and *a posteriori* approaches. The *a priori* methods, namely dietary indices, were hypothesis-driven and were used to quantified dietary quality according to dietary guidelines or certain types of diets (37). The DASH diet, as *a priori* dietary pattern, recommends higher intake of fruits, vegetables whole grains, poultry, fish, and nuts and restricts saturated fat, red meat, sweets beverages, and refined grains (6). Notably, a recent review on dietary intervention and blood pressure control showed that adherence to the DASH diet was significantly associated with lower level of blood pressure independently from the sodium intake (38). In addition to reducing blood pressure as the original purpose, adhering to the DASH diet has also been shown to be beneficial in reducing the risk of cardiovascular diseases (CVDs), diabetes, chronic kidney disease (CKD) and cancer (8, 12, 39). Benziger et al. (40) reported that adherence to healthy dietary patterns is an indispensable part of clinical guidelines to prevent and control non-communicable diseases, including several types of cancers. Further assurance was provided in two recent systematic review and meta-analyses, reporting that the adherence to the DASH diet was associated with reduced risk of colorectal cancer (12, 41). However, the effect of this pattern on breast cancer has been less studied. In the current meta-analysis, we found an inverse association between adherence to the DASH diet and risk of breast cancer. Similar to our findings, several studies have reported that “healthy” dietary patterns, which share some similar components with DASH diet, were inversely associated with the risk of breast cancer (42, 43). Besides, in a previous systematic review and meta-analysis of dietary patterns and breast cancer risk, Brennan et al. (4) also found that the “prudent/healthy” dietary pattern, characterized by high intake of fruit, vegetables, poultry, fish, low-fat dairy and whole grains, was associated with a 11% decreased risk of breast cancer. Also, the DASH dietary pattern which emphasizes higher intakes of fruits, vegetables, whole grains, nuts and legumes, moderate intake of low-fat dairy products, and lower intakes of red or processed meats, sugar-sweetened beverages and sodium (44), is in accordance with this “prudent/healthy” dietary patterns. This may justify its protective effect in reducing the risk of breast cancer. Meanwhile, the effect of the DASH diet on breast cancer may be related to their high concentration of some bioactive compounds, such as antioxidants (e.g., vitamin C, vitamin E, phenols, carotenoids, and flavonoids), minerals, dietary fiber, and folate. Researchers have also proposed several plausible explanations for the beneficial effect of DASH diet on breast cancer, although the exact mechanism is still unclear. First, antioxidants such as vitamin E and carotenoids can neutralize reactive oxygen species and protect against free radical damage involved in carcinogenes (45, 46). In addition, it is known that vitamin C can protect cells from oxidative DNA damage, thereby blocking carcinogenesis (47). Second, vegetables, fruits and whole grains, which are the most emphasized components of the DASH diet, are rich sources of dietary fiber. Findings from a previous systematic review and meta-analysis based on 16 prospective studies showed that high intake of dietary fiber was associated with a lower risk of breast cancer (48). Although the exact biologic mechanisms are unclear, experimental studies have shown that dietary fiber might reduce intestinal β-glucuronidase activity, which is necessary for hydrolysis of conjugated estrogens before absorption, thereby resulting in less reabsorption of estrogens (49). Besides, some studies also found that dietary fiber, especially soluble fiber may delay gastric emptying and increase small intestine transit time, thereby slowing glucose absorption, reducing insulin secretion and hyperinsulinemia (50), an important risk factor for breast cancer (51). Furthermore, high intake of dietary fiber may reduce the risk of overweight/obesity, which is an important risk factor for breast cancer (18). Third, fruits and vegetables are rich in folate. An up-to-date meta-analysis by Zeng et al. (52) reported that high consumption of folate, which is increased in the DASH diet, may have preventive effects against breast cancer in premenopausal women. Davis and Uthus (53) have reported that folate is necessary for synthesis of thymine and plays an important role in the synthesis, repair, and methylation of DNA, and thus preventing carcinogenesis. Fourth, nuts and legumes that provide a good source of polyphenols, including flavonoids and proanthocyanidins, are also recommended in the DASH diet. The growing body of scientific evidence indicates that flavonoids can prevent cancer through inactivation of carcinogens, inhibition of cell proliferation, enhancement of DNA repair processes, and reduction in oxidative stress (54). Besides, a previous study by Fillon (55) has also shown that these components might decrease the risk of cancer. Fifth, a lower consumption of red and processed meats is also recommended in the DASH dietary guideline. Red and processed meats are a major source of iron. It is mentioned in the previous studies that excessive consumption of iron could cause oxidative stress and endogenous formation of carcinogenic N-nitroso compounds (56). Also, processed meat often contains high concentrations of nitrates or nitrites, N-nitroso compounds and heterocyclic amines, which are thought to be carcinogenic (57). Finally, moderate intake of low-fat dairy products is promoted in the DASH diet. A recent meta-analysis of thirty-six observational studies revealed that consumption of dairy products was associated with a decreased risk of breast cancer (58). In brief, the aforementioned these mechanisms may account for the beneficial association between DASH diet and breast cancer.

### 4.1. Strengths and limitations

This systematic review and meta-analysis holds its own strengths and limitations. First, this is the first systematic review and meta-analysis so far assessing the relationship between adherence to the DASH diet and breast cancer risk. Our findings underline the importance of supporting the population in adhering to DASH diet for prevention of breast cancer. Second, the cases of breast cancer have been diagnosed through view of cancer registry or medical records or pathological records, avoiding misdiagnosis. Third, no signs of publication bias were evident in the funnel plot, and the statistical test for publication bias was non-significant. Fourth, the quality assessment showed that most studies included in this meta-analysis were of high quality. Lastly, the majority of the selected studies had adjusted for some potential confounders, which can reduce the effects of confounding factors. Despite these strengths, a number of limitations should be taken into account when interpreting our findings. First, 3 out of 11 studies included in this systematic review used the case-control design, which is more susceptible to recall and selection bias, than cohort design. In addition, owing to the observational nature of included studies, the possibility of residual bias from unmeasured, imprecisely measured, or unknown confounders remains. Hence, further prospective cohort studies or randomized controlled trials are needed to confirm the exact association between DASH diet and breast cancer. Second, the participants’ dietary intakes were self-reported through FFQs, which carried an inherent recall bias. At the same time, the levels of the highest and the lowest categories of DASH diet scores were inconsistent in the included studies, which might have attenuated the true association between adherence to the DASH diet and breast cancer risk. Third, only single time-point measurements of dietary patterns were provided in included studies, and these do not explain the changes in diet over time. Meanwhile, there was also an inconsistent adjustment for potential confounders in the included studies. As a result, the data included in our analyses may suffer from differing degrees of completeness and accuracy. Fourth, there was strong evidence for heterogeneity of results across the studies in our analyses. Subgroup analyses revealed that study design, country, menopausal status and comparison were the potential source of heterogeneity. Besides, it should be kept in mind that although the DASH diet was defined based on food groups in all included studies, the macro-nutrient composition of DASH diet was different between them, and this might explain the high heterogeneity found between included studies. Fifth, although DASH diet was associated with decreased risk of breast cancer, the results should be interpreted with caution. Because we could not determine and explain the source of between-study heterogeneity sufficiently. Finally, this study had a geographical restriction, as the majority of included studies came from the United States, where the dietary intakes were markedly different from the Asian countries. This leaded to a reduction in the heterogeneity of this meta-analysis and hence, further large prospective studies and randomized controlled trials are needed to confirm our findings in different regions and populations.

## 5. Conclusion

In conclusion, the current study revealed a significant inverse association between adherence to the DASH diet and the risk of breast cancer. Our findings add to the current evidence that healthy dietary pattern, like the DASH diet, could offer a practical strategy in the prevention of breast cancer. Further studies, particularly large prospective studies, are required to validate our findings in different geographic regions.

## Data availability statement

The original contributions presented in this study are included in the article/Supplementary material, further inquiries can be directed to the corresponding author.

## Author contributions

J-YZ designed the research. LS performed the systematic literature search, identified the studies meeting the inclusion criteria, extracted data from the included studies, and wrote the manuscript. Y-QH assessed the risk of bias of the included studies. QZ performed the statistical analysis. J-YZ and P-FZ assisted in the interpretation of the results and the revision of the manuscript. All authors had primary responsibilities for the final content and read and approved the final manuscript.
