# Supplementary material for: Adherence to the Dietary Approaches to Stop Hypertension diet reduces the risk of breast cancer: A systematic review and meta-analysis
Source: Front Nutr. 2023 Jan 9;9:1032654. doi: 10.3389/fnut.2022.1032654 (PMC9868726; doi:10.3389/fnut.2022.1032654)
Supplement: Supplementary file 1 [file Table_1.doc]

**Appendix1** DASH diet and breast cancer: Assessment of Study Quality

| **Studies** | **Selection** | | | |  | **Comparability** | |  | **Outcome** | | | **Score** |
| --- | --- | --- | --- | --- | --- | --- | --- | --- | --- | --- | --- | --- |
|  | 1 | 2 | 3 | 4 |  | 5A | 5B |  | 6 | 7 | 8 |  |
| Cohort |  |  |  |  |  |  |  |  |  |  |  |  |
| Izano et al 2013 [14] | * | * | * | * |  | * |  |  | * | * | * | ******** |
| Dela Cruz et al 2021 [15] | * | * | * | * |  | * |  |  | * | * | * | ******** |
| Petimar et al 2019 [16] | * | * | * | * |  | * | * |  | * | * | * | ********* |
| Ergas et al 2021[24] | * | * | * |  |  | * |  |  | * | * | * | ******* |
| Fung et al 2011[25] | * | * | * | * |  | * | * |  | * | * | * | ********* |
| Haridass et al 2018[26] | * | * | * | * |  | * | * |  | * | * | * | ********* |
| Wang et al 2020 [28] | * | * | * |  |  | * |  |  | * | * | * | ******* |
| Hirko et al 2016[29] | * | * | * | * |  | * | * |  | * | * | * | ********* |
| Case-control |  |  |  |  |  |  |  |  |  |  |  |  |
| Heidari et al 2020[13] | * |  | * | * |  | * |  |  | * | * |  | ****** |
| Soltani et al 2021[17] | * | * | * | * |  | * |  |  | * | * |  | ******* |
| Toorang et al 2021[27] | * |  | * | * |  | * |  |  | * | * |  | ****** |

*For case-control studies*,* 1 indicates cases independently validated; 2, cases are representative of population; 3, community controls; 4, controls have no history of blood pressure disease; 5A, study controls for age; 5B, study controls for additional factor(s); 6, ascertainment of exposure by blinded interview or record; 7, same method of ascertainment used for cases and controls; and 8, non response rate the same for cases and controls. For cohort studies, 1 indicates exposed cohort truly representative; 2, non exposed cohort drawn from the same community; 3, ascertainment of exposure; 4, outcome of interest not present at start; 5A, cohorts comparable on basis of age; 5B, cohorts comparable on other factor(s); 6, quality of outcome assessment; 7, follow-up long enough for outcomes to occur; and 8, complete accounting for cohorts.
