# Supplementary figures and images for: Adherence to the Dietary Approaches to Stop Hypertension diet reduces the risk of breast cancer: A systematic review and meta-analysis
Source: Front Nutr. 2023 Jan 9;9:1032654. doi: 10.3389/fnut.2022.1032654 (PMC9868726; doi:10.3389/fnut.2022.1032654)

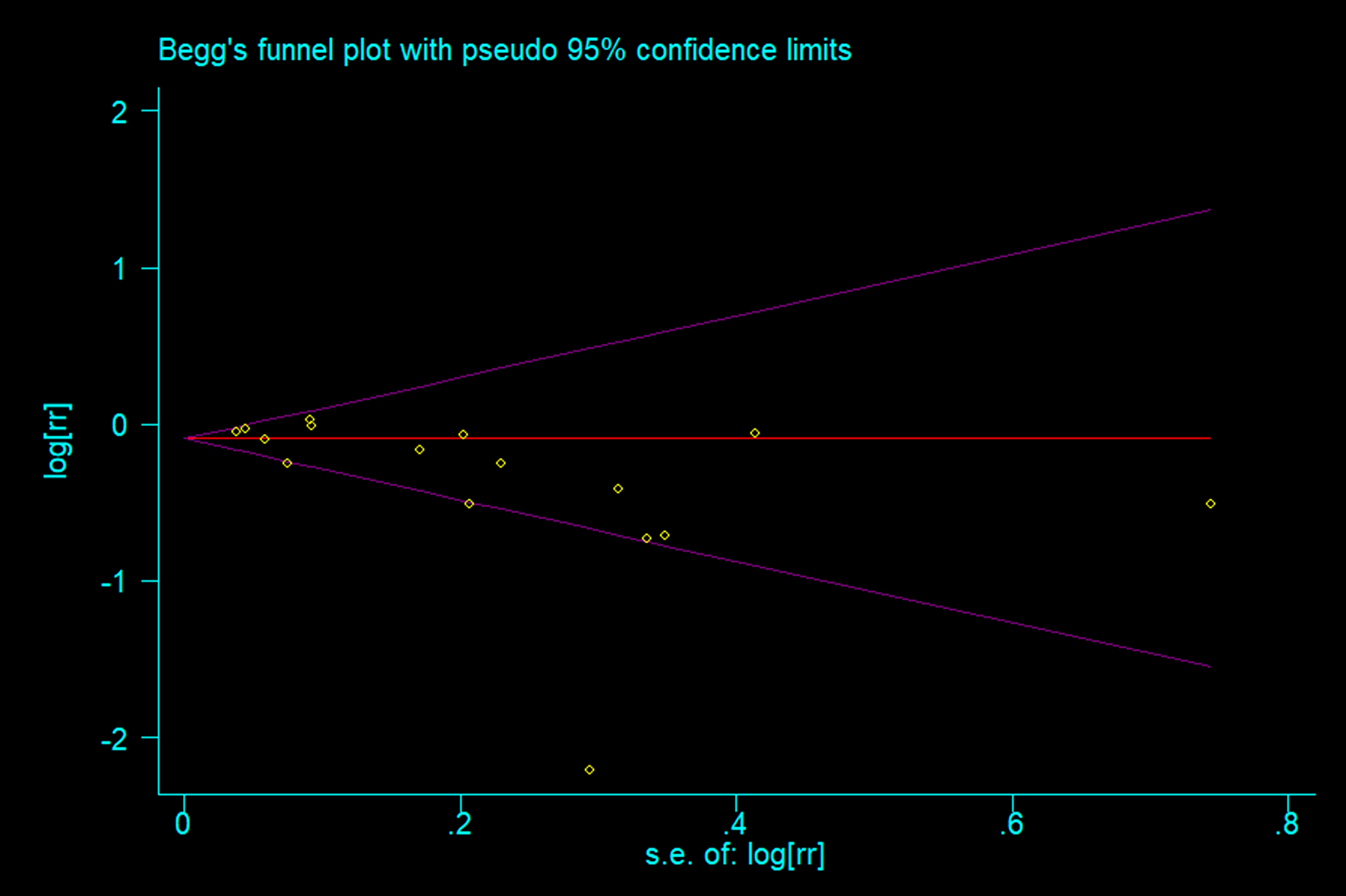

Supplement: Supplementary file 2 [file Image_1.TIF]

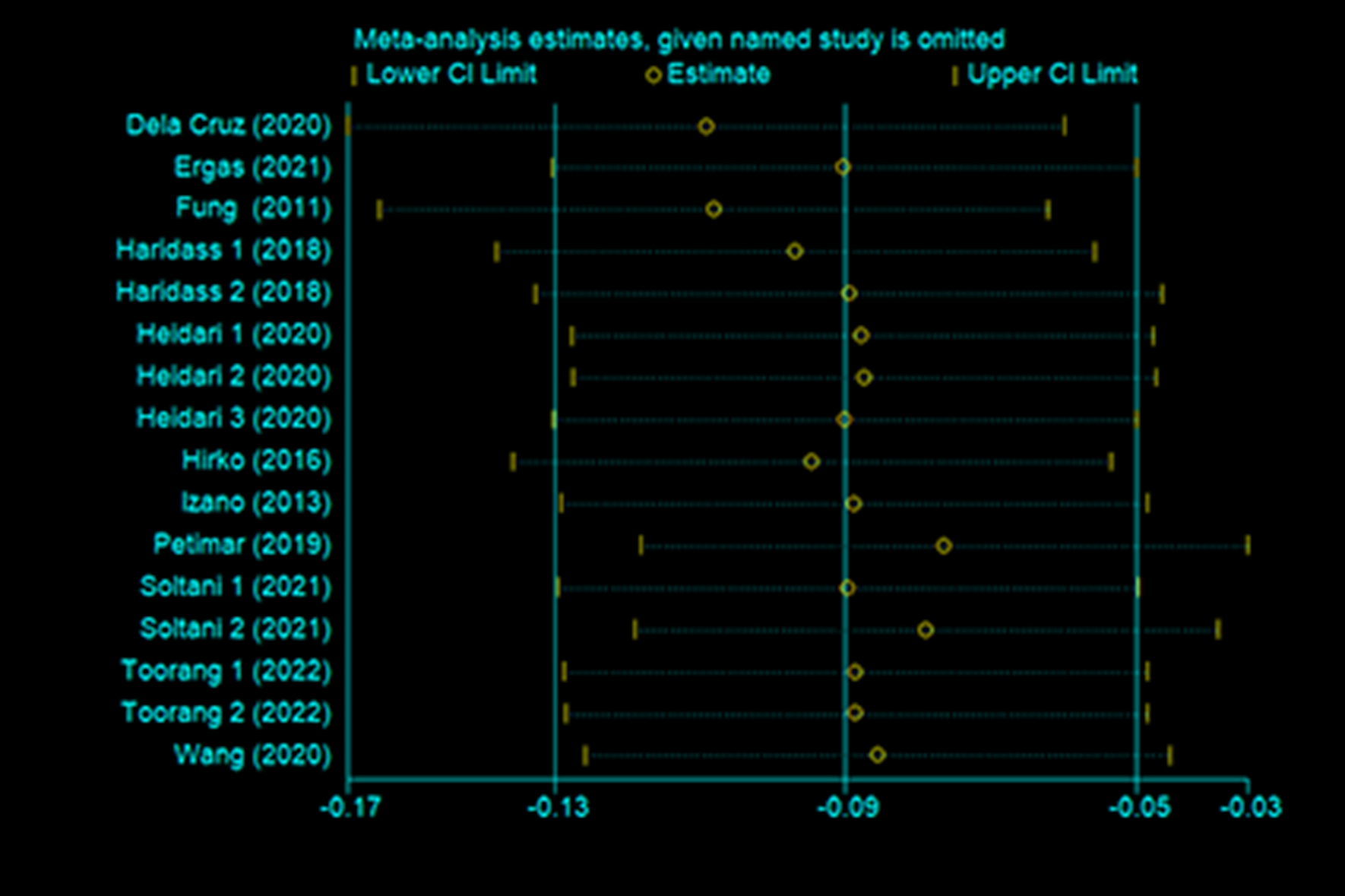

Supplement: Supplementary file 3 [file Image_2.TIF]
